# Supplementary material for: Residue Depletion Profile and Estimation of Withdrawal Period for Sulfadimethoxine and Ormetoprim in Edible Tissues of Nile Tilapia (Oreochromis sp.) on Medicated Feed
Source: Animals (Basel). 2023 Aug 3;13(15):2499. doi: 10.3390/ani13152499 (PMC10417122; doi:10.3390/ani13152499)
Supplement: Supplementary file 1 [file animals-13-02499-s001.zip › animals-2466855-supplementary.pdf]

SUPPLEMENTARY MATERIALS

Residue depletion profile and estimation of withdrawal period for sulfadimethoxine and ormetoprim in edible tissues of Nile tilapia (*Oreochromis* sp.) on medicated feed

Lucas Victor Pereira de Freitas[1,†], Carlos Augusto Alvarenga da Mota Júnior[1,†], Marina Alves Damaceno[1], Juliana Grell Fernandes Silveira[1], Ana Carolina Vellosa Portela[1], Sarah Chagas Campanharo[1], Agnaldo Fernando Baldo da Silva[1], Inácio Mateus Assane[2], Fabiana Pilarski[2], James Jacob Sasanya[3], and Jonas Augusto Rizzato Paschoal[1,*]

[1] School of Pharmaceutical Sciences of Ribeirão Preto, Department of Biomolecular Sciences, University of São Paulo (USP), Ribeirao Preto, SP 14040-900, Brazil

[2] Laboratory of Microbiology and Parasitology of Aquatic Organisms, Sao Paulo State University (Unesp), Aquaculture Center of Unesp, Jaboticabal, SP 14884-900, Brazil

[3] International Atomic Energy Agency, Vienna A-1400, Austria.

[*] Correspondence: paschoal@usp.br

[†] These authors contributed equally to this work

Table S1. The selected reaction monitoring (SRM) acquisition parameters for monitoring the target analytes.

Analyte	Molecular formula	Precursor ion (m/z)	Monitored transitions	Cone voltage (V)	Collision energy (eV)
SDM	$C_{12}H_{14}N_4O_4S$	311	311 > 156[1]	55	35
			311 > 245[2]	55	35
OMP	$C_{14}H_{18}N_4O_2$	275	275 > 259[1]	35	30
			275 > 123[2]	35	21
IS	$C_{10}H_{11}N_3O_3S$	254	254 > 156[1]	60	40
			254 > 98[2]	60	52

[1] Quantifier transition; [2] qualifier transition

SDM = sulfadimethoxine; OMP = ormetoprim; IS = Internal standard (sulfamethoxazole)

Table S2. Parameters of evaluation to assess the reliability of the analytical method for determination of SDM and OMP in fish feed.

**Parameter**	**OMP**	**SDM**
Linear range (μg mL^{-1})	0.2 – 1.0	1.0 – 5.0
Linearity (r)	0.9952 – 1.00	0.9981 – 1.00
Intra-day precision (CV, %, n = 3)		
OMP = 0.4; SDM = 2.0 (μg mL^{-1}, n = 3)	0.7	1.2
OMP = 0.6; SDM = 3.0 (μg mL^{-1}, n = 3)	2.2	0.9
OMP = 0.8; SDM = 4.0 (μg mL^{-1}, n = 3)	1.0	1.3
Inter-days precision (CV, %, n = 3)		
OMP = 0.4; SDM = 2.0 (μg mL^{-1}, n = 3)	2.2	1.8
OMP = 0.6; SDM = 3.0 (μg mL^{-1}, n = 3)	2.4	1.8
OMP = 0.8; SDM = 4.0 (μg mL^{-1}, n = 3)	2.3	0.8
Intra-day trueness (recovery, %, n = 3)		
OMP = 0.4; SDM = 2.0 (μg mL^{-1}, n = 3)	100.2	99.1
OMP = 0.6; SDM = 3.0 (μg mL^{-1}, n = 3)	102.5	101.1
OMP = 0.8; SDM = 4.0 (μg mL^{-1}, n = 3)	101.9	100.2
Inter-day trueness (recovery, %, n = 3)		
OMP = 0.4; SDM = 2.0 (μg mL^{-1}, n = 3)	98.7	100.2
OMP = 0.6; SDM = 3.0 (μg mL^{-1}, n = 3)	102.9	102.1
OMP = 0.8; SDM = 4.0 (μg mL^{-1}, n = 3)	101.7	100.7

SDM = sulfadimethoxine; OMP = ormetoprim; CV = coefficient of variation;

Table S3. Parameters of evaluation to assess the reliability of the analytical method for determination of SDM and OMP in fish fillet.

**Parameter**	**OMP**	**SDM**
Linear range (ng g^{-1})	20.0 – 260	20.0 – 260
Linearity (r)	0.997 – 0.99	0.996 – 1.00
Intra-day precision (CV, %, n = 3)		
20 (ng g^{-1}, n = 3)	2.3	8.4
140 (ng g^{-1}, n = 3)	3.6	3.8
260 (ng g^{-1}, n = 3)	0.8	1.9
Inter-days precision (CV, %, n = 3)		
20 (ng g^{-1}, n = 3)	2.5	5.1
140 (ng g^{-1}, n = 3)	0.9	3.3
260 (ng g^{-1}, n = 3)	0.9	4.7
Trueness (recovery, %, n = 3)		
OMP = 0.4; SDM = 2.0 (μg mL^{-1}, n = 3)	107	85.0
OMP = 0.6; SDM = 3.0 (μg mL^{-1}, n = 3)	95.0	106
OMP = 0.8; SDM = 4.0 (μg mL^{-1}, n = 3)	101	99.0
LOD (ng/g, n = 3)	1.0	3.0
LOQ (ng/g, n = 3)	20.0	20.0
LOQ precision (CV, %, n = 3)	2.3	8.4
LOQ trueness (recovery, %, n = 3)	107.2	85.4

SDM = sulfadimethoxine; OMP = ormetoprim; CV = coefficient of variation; LOD = limit of detection; LOQ = limit of quantification

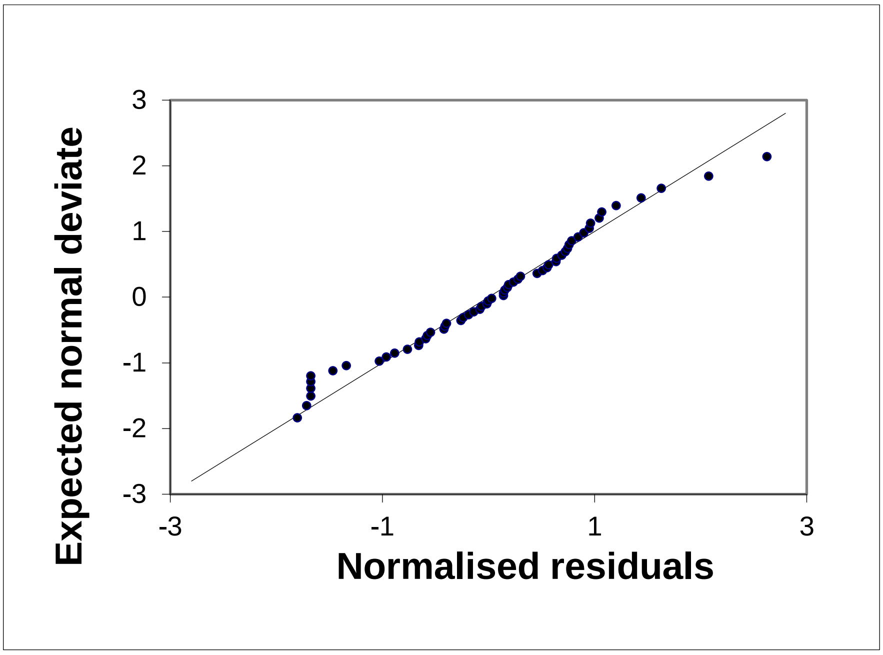

Fig. S1. Graph of the ordered normalized residuals versus their cumulative frequency, prepared on a normal probability scale.
